# Supplementary material for: Maternal Serum Concentrations of Per- and Polyfluoroalkyl Substances in Early Pregnancy and Small for Gestational Age in Southern Sweden
Source: Toxics. 2023 Sep 4;11(9):750. doi: 10.3390/toxics11090750 (PMC10534445; doi:10.3390/toxics11090750)
Supplement: Supplementary file 1 [file toxics-11-00750-s001.zip › toxics-2513920-supplementary.pdf]

Prof. Dr. med. H. Drexler
Institute and Outpatient Clinic for Occupational, Social and Environmental Medicine of the University of Erlangen-Nuremberg
Schillerstr.25 D-91054 Erlangen

Intercomparison programme 57, 2016 for toxicological analyses in biological materials

Inst. für Arbeits-, Sozial- und Umweltmedizin, Schillerstr. 25, 91054 Erlangen

Environmental Analysis Unit
Occupational and Environmental Medicine
Att: Thomas Lundh, Dr Med Sci , Skånes
University Hospital
22185 Lund
Sweden

Labor: 336

Erlangen, 2016/06/24

Youden Plot

No.	parameter		evaluation	your results	ref. value	tolerance range	unit
120	PFOA in serum	A:	+	6.02	5.95	4.90 - 7.00	µg/l
		B:	+	23.48	22.91	19.13 - 26.69	µg/l

PFOA in serum (Environmental medical field)

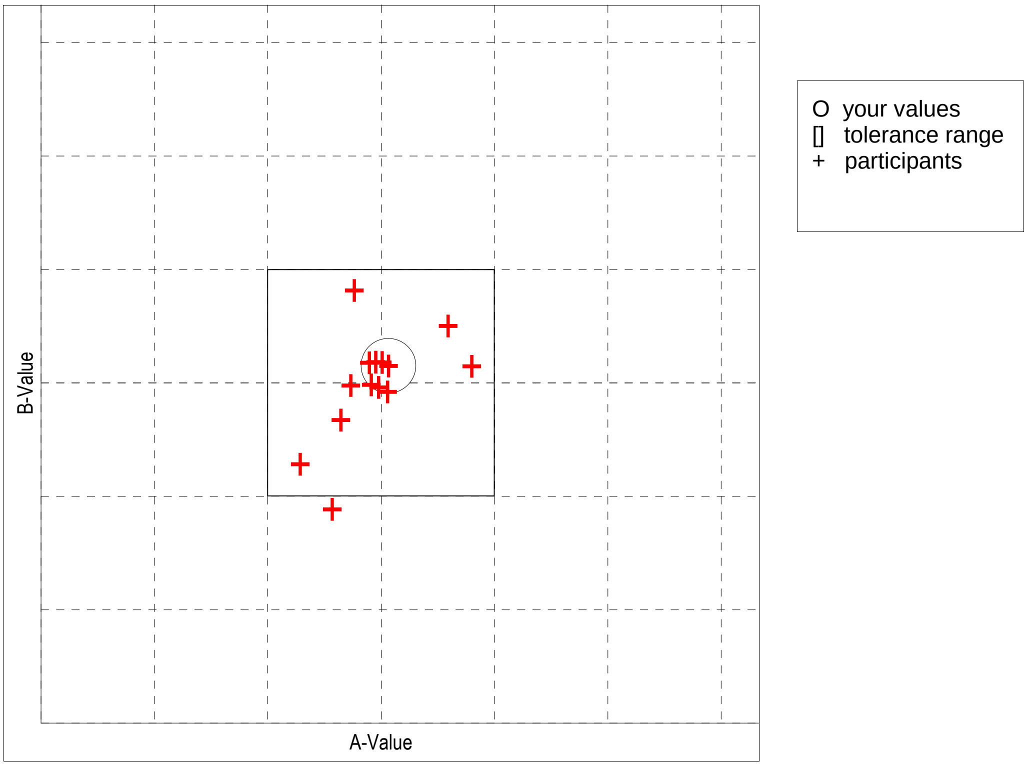

	A	B
number of participants	14	14
within 3-fold tolerance range	14	14
mean of 3-fold tolerance range	5.90	22.87
standard deviation 3-fold tolerance range	0.39	1.73
both values within tolerance range		13 Labs; (92.9%)

Prof. Dr. med. H. Drexler
Institute and Outpatient Clinic for Occupational, Social and Environmental Medicine of the University of Erlangen-Nuremberg
Schillerstr.25 D-91054 Erlangen

Intercomparison programme 57, 2016 for toxicological analyses in biological materials

Inst. für Arbeits-, Sozial- und Umweltmedizin, Schillerstr. 25, 91054 Erlangen

Environmental Analysis Unit
Occupational and Environmental Medicine
Att: Thomas Lundh, Dr Med Sci , Skånes
University Hospital
22185 Lund
Sweden

Labor: 336

Erlangen, 2016/06/24

Youden Plot

No.	parameter	evaluation		your results	ref. value	tolerance range	unit
121	PFOS in serum	A:	+	4.88	4.97	3.89 - 6.05	µg/l
		B:	+	38.17	39.73	32.26 - 47.20	µg/l

PFOS in serum (Environmental medical field)

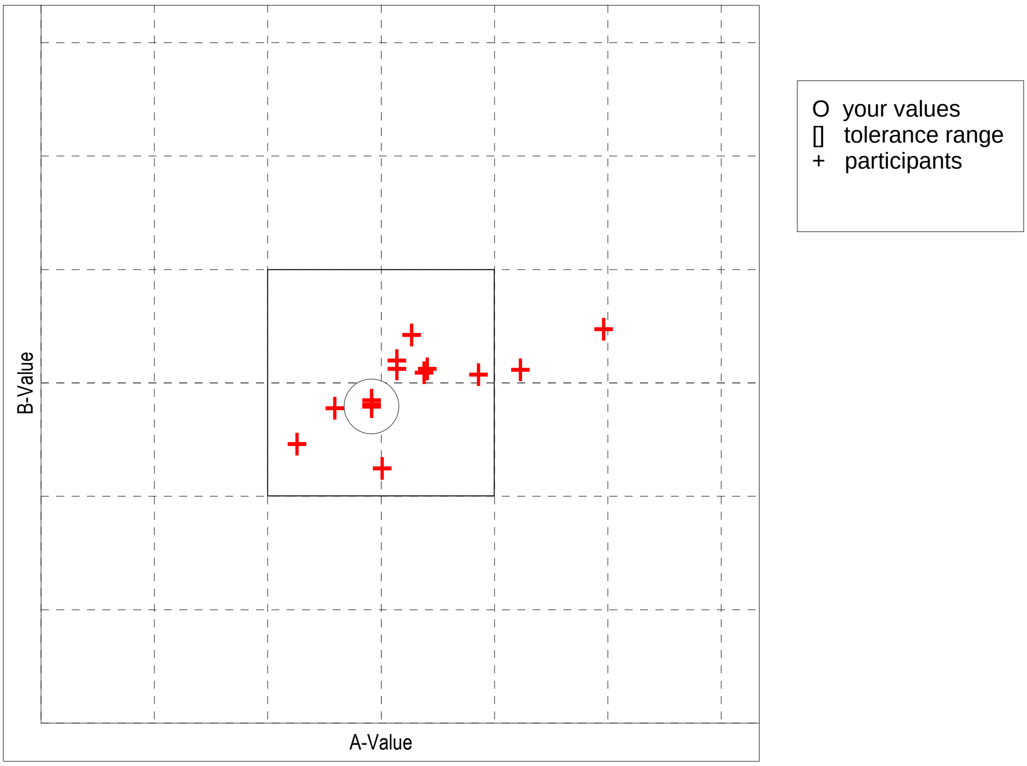

	A	B
number of participants	14	14
within 3-fold tolerance range	14	14
mean of 3-fold tolerance range	5.28	39.49
standard deviation 3-fold tolerance range	0.71	2.44
both values within tolerance range		12 Labs; (85.7%)

German External Quality Assessment Scheme

Intercomparison programme 57, 2016
for toxicological analyses in biological materials

Prof. Dr. med. H. Drexler

on behalf of the German Society for Occupational and Environmental Medicine .e.V

Schillerstr 25, D-91054 Erlangen

External Quality Control acc. to the Guidelines of the German Federal Medical Council

Teilnehmer: Environmental Analysis Unit 336
Occupational and Environmental Medicine
Att: Thomas Lundh, Dr Med Sci , Skånes University Hospital
22185 Lund
Sweden

Certificate

valid until July 31, 2017

This is to certify you participated in the intercomparison programme 57 / 2016 for occupational / environmental medical - toxicological analyses. In accordance with the guidelines issued by the German Federal Medical Council (Bundesärztekammer) of January 16th, 1987 and October 16th, 1987 and August 24th, 2001 on implementation of intercomparison programmes in the medical field you have fullfilled the requirements for the following parameters:

Environmental medical field

Pb in blood	Pt in urine	1-HP in urine
3-PBA in urine	Cotinine in urine	5-OH-MEHP in urine
MEHP in urine	PFOA in serum	PFOS in serum
MBzP in urine	Cu in urine	Sr in urine
Trichloropyridinol in urine	Sb in urine	Mo in urine
Sn in urine		

Erlangen, 2016/06/24

Prof. Dr. med. H. Drexler Prof. Dr. rer. nat. Th. Göen
